# Severe Adult HLH/MAS With SPTCL‐Like Panniculitis: A Phenotype‐Guided, Resource‐Adapted Therapeutic Strategy Without Cytotoxic Therapy

**DOI:** 10.1002/ccr3.72638

**Published:** 2026-04-29

**Authors:** Hatem Mousa Taha, Omar Marouf, Khaled Hatem Taha

**Affiliations:** ^1^ Faculty of Medicine and Health Sciences An‐Najah National University Nablus Palestine; ^2^ Department of Internal Medicine Palestine Medical Complex (PMC) Ramallah Palestine

**Keywords:** cyclosporine, cytokine storm, hemophagocytic lymphohistiocytosis, hyperferritinemia, intravenous immunoglobulin, macrophage activation syndrome, panniculitis, resource‐limited setting, SPTCL, therapeutic plasma exchange

## Abstract

Hemophagocytic lymphohistiocytosis (HLH) and macrophage activation syndrome (MAS) are life‐threatening hyperinflammatory conditions with heterogeneous triggers and overlapping clinical phenotypes. Diagnostic uncertainty is particularly challenging in resource‐limited settings, where advanced molecular and immunophenotypic investigations are not readily available. A 31‐year‐old woman presented with recurrent febrile episodes, trilineage cytopenias, hyperferritinemia (peak 16,970 ng/mL [reference: < 200 ng/mL]), hypofibrinogenemia, and progressive necrotic panniculitis plaques involving both lower extremities. Histopathological evaluation revealed lobular panniculitis with adipocyte necrosis and absence of vasculitis. While these findings raised strong concern for subcutaneous panniculitis‐like T‐cell lymphoma (SPTCL), definitive diagnosis was precluded by the absence of immunophenotyping, T‐cell receptor clonality studies, and HAVCR2 mutation testing—all unavailable in our setting. The HScore was 216 (> 99% probability of HLH/MAS). A striking feature was the persistent dissociation between markedly elevated ferritin and low C‐reactive protein (CRP) values (ratio < 0.002), consistent with a non‐IL‐6‐dominant, interferon‐γ–driven hyperinflammatory state. Based on this phenotype, a sequential treatment strategy combining therapeutic plasma exchange (TPE), intravenous immunoglobulin (IVIG), and cyclosporine A (CyA) was implemented without cytotoxic chemotherapy. The patient demonstrated significant clinical and biochemical improvement, with resolution of cytopenias and progressive decline in ferritin. This case highlights the diagnostic overlap between autoimmune panniculitis, SPTCL, and HLH/MAS, and underscores the importance of phenotype‐guided management in complex hyperinflammatory syndromes. It further illustrates that effective treatment can be achieved without cytotoxic therapy in selected patients, even in the absence of definitive molecular diagnosis.

## Key Clinical Message

Severe HLH/MAS may mimic subcutaneous panniculitis‐like T‐cell lymphoma (SPTCL), particularly in resource‐limited settings. CRP–ferritin dissociation may indicate a non‐IL‐6–driven hyperinflammatory state consistent with macrophage activation. Phenotype‐guided therapy combining therapeutic plasma exchange, intravenous immunoglobulin, and cyclosporine can achieve effective disease control even without definitive molecular diagnosis or cytotoxic treatment.

## Graphical abstract

Graphical abstract illustrating the phenotype‐guided therapeutic approach in severe adult HLH/MAS with SPTCL‐like panniculitis, demonstrating clinical and biochemical response following sequential treatment with therapeutic plasma exchange, intravenous immunoglobulin, and cyclosporine.

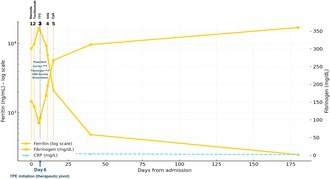

## 1 Background

Hyperferritinemic syndromes within the HLH/MAS spectrum are characterized by uncontrolled macrophage and T‐cell activation, excessive cytokine release, immune dysregulation, cytokine storm, multi organ dysfunction, and consumptive coagulopathy [1, 2, 3, 4]. Adult presentations often overlap autoimmune‐associated MAS, infection‐triggered HLH, and autoinflammatory states, with heterogeneous phenotypes that do not fit rigid diagnostic categories [2, 3].

In highly resourced settings, IFN‐γ‐pathway blockade and Janus kinase (JAK) inhibition have expanded therapeutic options [5, 6], whereas in many low‐ and middle‐income countries, lack of cytokine panels and repeated biologics necessitates reliance on pragmatic bedside surrogates [2, 7]. Importantly, malignancy‐associated HLH—particularly lymphoma‐driven presentations—must always be considered in adult patients, as treatment approach differs substantially from autoimmune‐associated HLH.

This report, prepared in accordance with CARE guidelines [8], describes a feasibility case of sequential non‐cytotoxic management in severe adult HLH/MAS with panniculitis features strongly overlapping subcutaneous panniculitis‐like T‐cell lymphoma (SPTCL) in a resource‐limited setting.

## 2 Case Presentation

A previously independent 31‐year‐old woman presented with 1 month of persistent high‐grade fever (39°C–40°C), progressive painful necrotic ulcers over both shins, severe fatigue, anorexia, and approximately 8 kg unintentional weight loss. Over the preceding 3 years she had recurrent tender subcutaneous plaques on both shins. Skin biopsy performed 18 months prior demonstrated lobular panniculitis with dense neutrophilic infiltrate, necrosis of adipocytes, and absence of vasculitis, consistent with sterile neutrophilic panniculitis. The lesions were partially responsive to intermittent oral prednisolone. There was no history of malignancy, diagnosed connective tissue disease, immunodeficiency, or consanguinity.

On admission she was acutely ill, febrile (39.4°C), tachycardic (118 beats/min), and mildly hypotensive. Examination revealed hepatosplenomegaly (liver and spleen palpable several centimeters below costal margin) and extensive necrotic panniculitis plaques over both shins, measuring up to 12 × 8 cm, with violaceous borders, central black eschar, and surrounding induration. The panniculitis plaques and ulcers represent a spectrum of the same pathological process: lesions initially presenting as indurated panniculitis plaques progressed to frank ulceration with central eschar formation, and these terms therefore denote the same pathological continuum at different stages of disease evolution rather than separate distinct entities. The dynamic cutaneous evolution of these lesions is illustrated in Figure 2. There was no clinically evident lymphadenopathy, synovitis, pharyngitis, typical salmon‐pink rash, or neurological deficit.

## 3 Investigations

**FIGURE 1:**
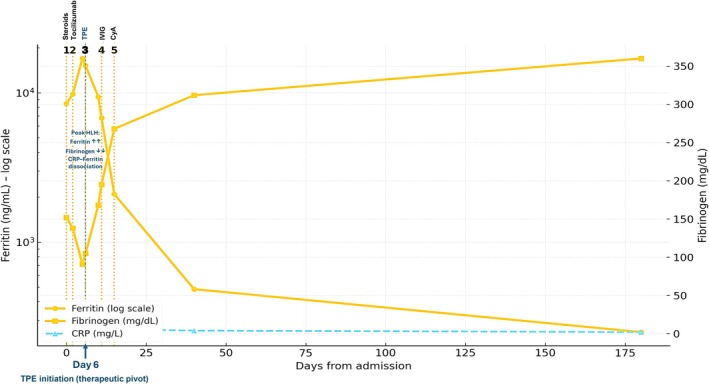
Temporal kinetics of ferritin, fibrinogen, and C‐reactive protein (CRP) with annotation of key therapeutic interventions in adult HLH/MAS. Serial trajectories of ferritin (log scale; left *y*‐axis), fibrinogen (right *y*‐axis, mg/dL), and CRP (right *y*‐axis, mg/L) from admission to 6‐month follow‐up. The early disease phase demonstrates a classic inverse ferritin–fibrinogen relationship, with CRP remaining disproportionately low relative to ferritin, consistent with CRP–ferritin dissociation. Peak disease activity is reached on approximately day 5. Dashed vertical lines indicate the timing of key therapeutic interventions: Line 1 (Day 0) = initiation of IV methylprednisolone; Line 2 (Day 2) = tocilizumab single dose; Line 3 (Day 6, highlighted as TPE initiation therapeutic pivot) = initiation of therapeutic plasma exchange; Line 4 (Days 11–15) = IVIG; Line 5 (Day 15) = cyclosporine A commenced. The marked decline in ferritin following the Day 6 pivot reflects the clinical turning point. Subsequent treatment is associated with progressive decline in ferritin and recovery of fibrinogen, reflecting resolution of the hyperinflammatory state. CRP remained relatively low throughout despite extreme hyperferritinemia, supporting a non‐IL‐6‐dominant inflammatory phenotype. These findings are presented as phenotype‐consistent observations.

At presentation, laboratory evaluation demonstrated marked systemic hyperinflammation with trilineage cytopenias (WBC 1.9 × 10^9^/L [reference: 4.0–11.0 × 10^9^/L]; hemoglobin 8.2 g/dL [reference: 12.0–16.0 g/dL]; platelet nadir 50 × 10^9^/L [reference: 150–400 × 10^9^/L]), extreme hyperferritinemia (8450 ng/mL on admission, rising to peak 16,970 ng/mL on day 5 [reference: 11–307 ng/mL]), and consumptive coagulopathy with hypofibrinogenemia (152 mg/dL on admission, nadir 91 mg/dL on day 5 [reference: 200–400 mg/dL]). C‐reactive protein (CRP) was only moderately elevated throughout (28 mg/L at admission, declining to 15 mg/L at peak ferritin [reference: < 5 mg/L]), producing a persistently low CRP‐ferritin ratio (< 0.002, specifically 0.0009 at peak disease activity). This CRP–ferritin dissociation is consistent with a non‐interleukin‐6 (IL‐6)‐driven hyperinflammatory state, as described in macrophage activation syndrome (MAS) and interferon‐γ (IFN‐γ)‐dominant conditions [9]. Peak disease activity (day 5), characterized by extreme hyperferritinemia and hypofibrinogenemia, is clearly demonstrated in Figure 1.

Serum triglycerides were elevated at 2.8 mmol/L (reference: < 1.7 mmol/L), and liver enzymes showed moderate transaminitis (peak alanine aminotransferase [ALT]/aspartate aminotransferase [AST] 112/97 U/L [reference: < 40 U/L]) with hypoalbuminemia, while renal function remained preserved. Lactate dehydrogenase (LDH) was elevated at 487 U/L (reference: 120–246 U/L). Using the HScore for reactive hemophagocytic syndromes [1], the patient scored 216 points, corresponding to > 99% probability of HLH/MAS.

Diagnostic limitations: Specialized HLH assays including soluble interleukin‐2 receptor (sCD25), NK‐cell activity, and cytokine panels (IL‐6, IFN‐γ, IL‐18, tumor necrosis factor‐alpha [TNF‐α]) were not available in this setting. Advanced molecular testing including HAVCR2 mutation analysis, T‐cell receptor (TCR) clonality studies, and comprehensive immunophenotyping were also unavailable, representing a critical diagnostic limitation as discussed below. Diagnostic probability therefore relied on clinical features, routine laboratory kinetics, and HScore‐based assessment.

Bone marrow examination, performed after exposure to high‐dose corticosteroids and tocilizumab, showed hypercellular marrow with preserved trilineage hematopoiesis and no overt hemophagocytosis or malignancy. This finding was interpreted in context given the known limited sensitivity of hemophagocytosis in adult HLH/MAS following immunomodulatory therapy [10, 11].

Contrast‐enhanced CT chest/abdomen/pelvis demonstrated hepatosplenomegaly without lymphadenopathy, masses, or organ infiltration.

Microbiological assessment of necrotic panniculitis ulcers yielded *Pseudomonas aeruginosa* sensitive to piperacillin‐tazobactam, which was administered intravenously for 14 days. Repeat wound cultures on days 10 and 14 yielded no growth, confirming microbiological clearance. Blood cultures remained sterile throughout.

Extended autoimmune screening (antinuclear antibody [ANA], extractable nuclear antigen [ENA], anti‐double‐stranded DNA [anti‐dsDNA], rheumatoid factor [RF], anti‐cyclic citrullinated peptide [anti‐CCP], antineutrophil cytoplasmic antibody [ANCA]) was negative. Complement levels (C3, C4) were normal.

Serial laboratory parameters and treatment timeline are summarized in Table 1. The temporal evolution of ferritin, fibrinogen, and CRP, with annotation of key therapeutic interventions, is illustrated in Figure 1. The clinical turning point, marked by initiation of therapeutic plasma exchange on day 6, is highlighted by the pivotal dashed line in Figure 1.

**TABLE 1:** Serial laboratory parameters and treatment timeline.

Parameter	Reference range	Day 0	Day 5 (★Peak)	Day 15	Day 40	Day 180	Unit
Ferritin	11–307	8450	16,970	4200	485	< 300	ng/mL
CRP	< 5	28	15	18	6	< 3	mg/L
CRP:Ferritin ratio	—	0.0033	0.0009	0.0043	0.012	> 0.01	—
Fibrinogen	200–400	152	91	178	312	> 350	mg/dL
WBC	4.0–11.0	1.9	1.4	2.1	5.8	6.9	×10^9^/L
Hemoglobin	12.0–16.0	8.2	7.6	8.4	10.8	12.4	g/dL
Platelets	150–400	80	50	72	168	224	×10^9^/L
Triglycerides	< 1.7	2.8	3.1	2.4	1.6	1.3	mmol/L
ALT/AST	< 40/< 40	95/81	112/97	78/65	42/38	Normal	U/L
LDH	120–246	487	540	380	195	Normal	U/L
Treatment milestones	—	IV methylprednisolone; Tocilizumab ×1	TPE × 5 (Days 6–10)	IVIG (Days 11–15); CyA started Day 15	CyA trough 138 ng/mL	CyA maintenance; remission sustained	—

*Note:* ★: Disease nadir. Red shading: nadir values; amber: near‐nadir; green: remission. Reference ranges apply to adult females. Detailed laboratory trends and therapeutic timelines are complemented by Figure 1.

Abbreviations: ALT, alanine aminotransferase; AST, aspartate aminotransferase; CRP, C‐reactive protein; CyA, cyclosporine A; IV, intravenous; IVIG, intravenous immunoglobulin; LDH, lactate dehydrogenase; TPE, therapeutic plasma exchange; WBC, white blood cell count.

## 4 Differential Diagnosis

Differential considerations included adult‐onset Still's disease with MAS, infection‐driven cytokine storm, malignancy‐associated HLH, VEXAS‐like syndrome, and primary (familial) HLH. A structured summary of the differential diagnostic considerations, including supporting and opposing features and key investigations, is provided in Table S1. Critically, subcutaneous panniculitis‐like T‐cell lymphoma (SPTCL) was identified as a key differential diagnosis and is discussed in detail below.

SPTCL as a major differential diagnosis: The clinical and histopathological profile of this case—extensive necrotic panniculitis plaques on both lower extremities, lobular panniculitis with adipocyte necrosis, and absence of vasculitis on biopsy—demonstrates substantial overlap with SPTCL. SPTCL is a well‐recognized cause of secondary HLH and should be considered in any adult presenting with panniculitis and hyperinflammatory features [12, 13].

Other malignancy‐associated HLH: In adults presenting with HLH, malignancy‐associated aetiologies are critical to consider. Beyond SPTCL, intravascular large B‐cell lymphoma (IVLBCL) and angioimmunoblastic T‐cell lymphoma (AITL) may present with nonspecific systemic and cutaneous inflammatory features and can be diagnostically challenging even with extensive workup. These diagnoses must be systematically considered and excluded, as management strategies differ substantially from autoimmune‐associated HLH.

Absence of classic Still's disease features (salmon‐pink rash, arthritis, pharyngitis), negative lupus serology, lack of malignancy on imaging and bone marrow, resolution of documented bacterial infection under targeted therapy yet ongoing hyperferritinemia and coagulopathy, and the presence of chronic neutrophilic panniculitis collectively favoured secondary HLH/MAS with strong SPTCL overlap rather than autoimmune panniculitis alone. Primary (genetic) HLH was considered less likely given adult age of onset, absence of family clustering, and response to non‐etoposide‐based immunomodulation, although genetic testing was not feasible.

## 5 Treatment and Outcome

High‐dose intravenous methylprednisolone was initiated on admission at approximately 1 mg/kg/day and continued for 14 days, resulting in only transient clinical improvement. Persistent fever, progressive cytopenias, and rising ferritin prompted administration of a single dose of tocilizumab (8 mg/kg intravenously) on day 2.

Within 72 h, despite appropriate antimicrobial therapy, the patient exhibited biochemical and clinical deterioration, with escalating ferritin (peak 16,970 ng/mL on day 5), worsening hypofibrinogenemia (nadir 91 mg/dL), and continued cytopenias. We emphasize that a single 8 mg/kg dose of tocilizumab may be insufficient to assess the IL‐6 pathway contribution to this patient's inflammatory state, and we describe this as “progression despite single‐dose IL‐6 blockade” rather than definitive IL‐6 blockade failure.

### 5.1 Sequential Non‐Cytotoxic Rescue Strategy

In the setting of objective disease progression, marked CRP–ferritin dissociation, evolving consumptive coagulopathy, and restricted access to repeated biologic therapy, a non‐cytotoxic rescue strategy was adopted.

Therapeutic plasma exchange (TPE) was initiated on day 6, with five consecutive daily sessions (approximately 1.0–1.2 plasma volumes exchanged per session using fresh frozen plasma replacement). As illustrated in Figure 1, the clinical turning point following TPE initiation is clearly reflected in the progressive decline of ferritin and concurrent normalization of fibrinogen. Following completion of TPE, high‐dose intravenous immunoglobulin (IVIG) was administered at 0.4 g/kg/day for 5 days (total dose 2 g/kg). Oral cyclosporine A (CyA) was started on day 15 at approximately 3 mg/kg/day in divided doses. Therapeutic trough levels were monitored: 175–195 ng/mL during acute phase (days 15–30), 138 ng/mL at day 40, and 120–145 ng/mL during maintenance.

**FIGURE 2:**
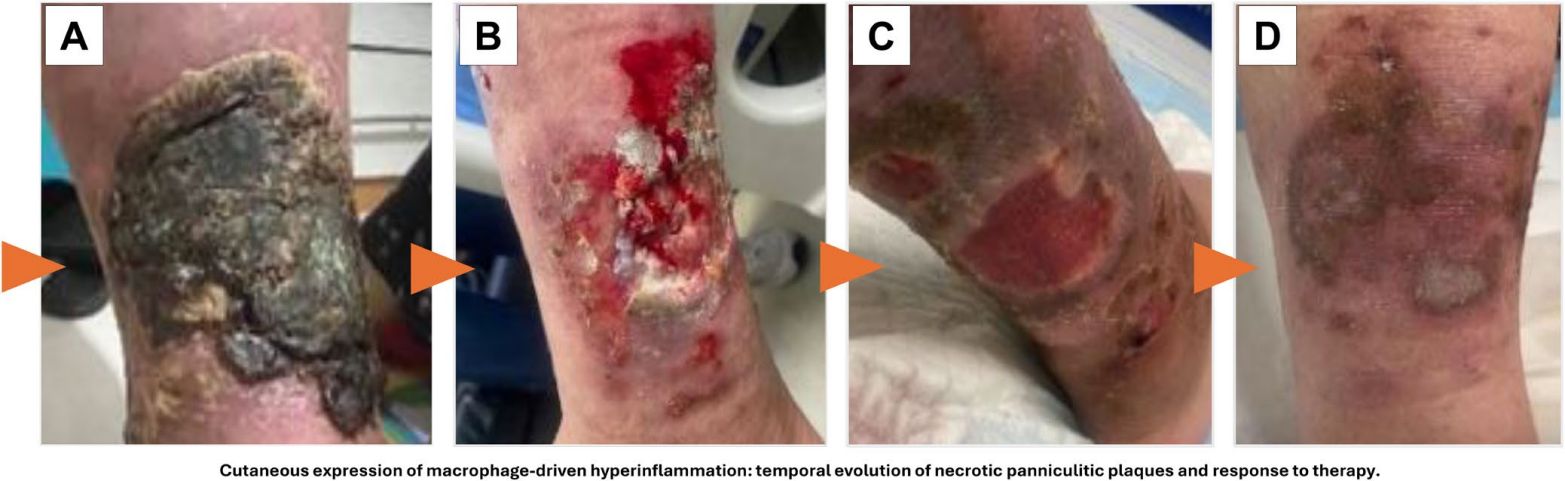
Cutaneous expression of macrophage‐driven hyperinflammation: Temporal evolution of necrotic panniculitis plaques and response to therapy. Serial clinical photographs illustrating the dynamic cutaneous manifestation of systemic hyperinflammation. Orange arrows indicate chronological progression. These cutaneous changes parallel the systemic hyperinflammatory trajectory demonstrated by biomarker kinetics in Figure 1. Panel (A) (Initial presentation): Extensive necrotic panniculitis plaque with thick adherent eschar, violaceous inflammatory borders, and central coagulative necrosis, consistent with severe adipose tissue injury. Lesions measured up to approximately 12 × 8 cm at peak disease. Panel (B) (Inflammatory peak, ∼day 5): Maximal lesion activity characterized by ulceration, progressive necrotic tissue breakdown, inflammatory exudate, and expanding erythematous–violaceous margins reflecting intense local inflammatory injury. Panel (C) (Repair phase, days 20–40): Progressive tissue recovery with granulation tissue formation, contraction of the ulcer base, and advancing re‐epithelialization following initiation of immunomodulatory therapy. Panel (D) (Late outcome, 6‐month follow‐up): Complete clinical resolution with residual post‐inflammatory hyperpigmentation and scar remodeling. Note formation of new smaller panniculitis nodules indicating residual but controlled disease activity, resolved on continued maintenance cyclosporine.

Clinical defervescence and progressive healing of panniculitis plaques and ulcers were observed within 10–14 days of initiating the TPE‐IVIG‐CyA sequence. The dynamic cutaneous evolution—from initial necrotic eschar through healing to complete resolution—is documented in Figure 2. These cutaneous changes (Figure 2) parallel the systemic hyperinflammatory trajectory demonstrated by biomarker kinetics in Figure 1. By day 40, the patient was afebrile, ferritin had fallen to 485 ng/mL, fibrinogen had normalized (312 mg/dL), blood counts had recovered, and necrotic skin lesions had fully re‐epithelialized. Corticosteroids were gradually tapered and discontinued. At 6 and 8 months, the patient remained in sustained remission on low‐dose cyclosporine monotherapy (~2 mg/kg/day).

## 6 Discussion

This case illustrates a severe hyperinflammatory syndrome consistent with HLH/MAS, occurring in the context of chronic relapsing panniculitis with significant diagnostic complexity, most notably a strong clinicopathological overlap with subcutaneous panniculitis‐like T‐cell lymphoma (SPTCL).

### 6.1 SPTCL as a Critical Diagnostic Consideration

The presence of extensive necrotic panniculitis plaques involving the lower extremities, together with histopathological findings of lobular panniculitis, adipocyte necrosis, and absence of vasculitis, demonstrates substantial overlap with SPTCL phenotypes. SPTCL is a rare cytotoxic T‐cell lymphoma that characteristically involves the subcutaneous tissue and is a well‐recognized cause of secondary HLH [12]. In a case series of 16 patients, individuals typically presented with nodular or plaque‐like lesions affecting the lower extremities, which is precisely the pattern observed in the current case [13].

However, several features argue against a definitive diagnosis of SPTCL: (1) the prolonged relapsing–remitting course without progression to overt lymphoma over the documented follow‐up period; (2) the absence of documented T‐cell clonality, malignant cytologic features, or CD8+/αβ+ T‐cell rim pattern on immunohistochemistry; (3) the sustained clinical and biochemical response to immunomodulatory rather than cytotoxic or lymphoma‐directed therapy; and (4) the benign bone marrow findings. Nevertheless, we cannot exclude SPTCL with certainty given the absence of advanced molecular diagnostics.

We therefore interpret this case as residing at the interface between immune‐mediated panniculitis disorder and SPTCL‐spectrum disease, with secondary HLH/MAS phenotype. This is an important and clinically challenging diagnostic overlap, particularly in resource‐limited settings where confirmatory testing is unavailable. Pathology re‐review with immunophenotyping was strongly considered; however, advanced immunohistochemical and molecular analyses—including CD8 immunostaining, TCR clonality studies, and HAVCR2 sequencing—were not available in our setting, representing a fundamental limitation of this report.

### 6.2 HAVCR2 Mutation Testing: A Critical Unmet Diagnostic Need

HAVCR2 mutation testing is strongly recommended in suspected SPTCL, and its absence in this case represents an important limitation. Germline mutations in HAVCR2, encoding the immune checkpoint protein T‐cell immunoglobulin and mucin domain‐containing protein 3 (TIM‐3), have been identified in approximately 60% of SPTCL cases and are increasingly recognized as key pathogenic drivers of immune dysregulation and HLH susceptibility [12, 13].

Mutation patterns may also vary by ethnicity: the p.Tyr82Cys variant is more commonly observed in East Asian populations, whereas the p.Ile97Met mutation is more frequently reported in Caucasian patients [12]. Critically, HAVCR2‐mutated SPTCL has demonstrated exquisite sensitivity to JAK inhibition, which has important therapeutic implications [14]. In this case, HAVCR2 testing and T‐cell receptor clonality studies were not performed due to resource limitations, and therefore an underlying SPTCL‐spectrum disorder cannot be definitively excluded. Future evaluation with HAVCR2 mutation analysis and TCR clonality studies would be essential to further clarify the underlying disease biology and to determine eligibility for JAK inhibitor therapy.

### 6.3 Malignancy‐Associated HLH: Expanding the Differential

In adult patients presenting with HLH, underlying malignancies must be systematically considered. Beyond SPTCL, intravascular large B‐cell lymphoma (IVLBCL) and angioimmunoblastic T‐cell lymphoma (AITL) are diagnostically challenging conditions that may present with nonspecific systemic and inflammatory features, skin manifestations, and hyperferritinemia, and can be missed on standard imaging and bone marrow examination. High clinical suspicion, repeated histopathological sampling, and comprehensive immunophenotyping are required. This case highlights the diagnostic continuum between inflammatory panniculitis, lymphoma‐associated HLH, and cytokine‐driven hyperinflammatory syndromes. A structured differential diagnostic framework supporting this interpretation is summarized in Table S1.

### 6.4 CRP–Ferritin Dissociation as a Phenotypic Biomarker

A central feature of this case is the pronounced dissociation between markedly elevated ferritin levels and persistently low CRP. As demonstrated in Figure 1, the inverse relationship between ferritin and fibrinogen, together with CRP–ferritin dissociation, supports a macrophage‐driven hyperinflammatory phenotype. CRp values remained low (15–28 mg/L) despite extreme hyperferritinemia (up to 16,970 ng/mL), yielding a CRP:ferritin ratio of 0.0009 at peak disease activity. This pattern reflects a non‐IL‐6‐dominant hyperinflammatory state and is more consistent with IFN‐γ–driven macrophage activation, as described in MAS and HLH [9]. Such CRP–ferritin dissociation contrasts with typical sepsis‐mediated inflammation, in which CRP is often markedly elevated and provides a clinically useful surrogate marker in settings where advanced cytokine profiling is unavailable. Detailed laboratory values at each time point are provided in Table 1, complementing the visual trends shown in Figure 1.

### 6.5 Rationale for Sequential Non‐Cytotoxic Strategy

The adoption of a fully non‐cytotoxic sequence—TPE, IVIG, and CyA—was guided by disease severity, evolving coagulopathy, profound cytopenias, recent Gram‐negative infection, and limited access to repeated biologics or etoposide‐based protocols. TPE allowed rapid debulking of circulating ferritin, inflammatory mediators, and procoagulant factors [15, 16, 17]. IVIG consolidated immune downregulation during transition out of the cytokine‐storm phase [18, 19]. Cyclosporine A provided sustained T‐cell inhibition via calcineurin‐NFAT pathway blockade, with relevance to the potential SPTCL‐HLH overlap given its immunomodulatory effects on the T‐cell–macrophage axis [20].

Interleukin‐1 blockade (anakinra) and JAK inhibition are recognized rescue options in refractory adult HLH/MAS [21]. In our setting, anakinra was not immediately accessible, and JAK inhibition posed cost and infection‐risk concerns given profound cytopenias and recent wound infection. Notably, if HAVCR2 mutation testing had been available and confirmed a SPTCL‐spectrum diagnosis, JAK inhibitor therapy would have been the preferred approach given its reported exquisite efficacy in HAVCR2‐mutated disease [14].

### 6.6 Fundamental Limitations

This case carries fundamental limitations that must be stated clearly. First, causality cannot be established from a single case with multiple concurrent interventions. Second, single‐dose tocilizumab may be insufficient to assess IL‐6 pathway contribution. Third, the absence of HAVCR2 testing, TCR clonality studies, and comprehensive immunophenotyping means an underlying SPTCL‐spectrum disorder cannot be definitively excluded. Fourth, 6–8 months follow‐up, while encouraging, is insufficient to confirm long‐term durability or exclude late relapse or lymphomatous transformation. Fifth, this case cannot establish superiority, non‐inferiority, or equivalence to standard HLH/MAS regimens.

## 7 Learning Points

1. Adult HLH/MAS may overlap significantly with SPTCL, and this critical differential must be explicitly considered in any adult presenting with panniculitis lesions and hyperinflammatory features.
2. HAVCR2 mutation testing and TCR clonality studies are strongly recommended in suspected SPTCL; their absence in resource‐limited settings should be acknowledged as a diagnostic limitation. HAVCR2‐mutated SPTCL has demonstrated exquisite sensitivity to JAK inhibition, underscoring the therapeutic relevance of molecular characterization [14].
3. CRP–ferritin dissociation may offer a pragmatic bedside surrogate for inflammatory phenotype classification (non‐IL‐6‐driven versus IL‐6‐driven hyperinflammation) in resource‐limited settings.
4. Sequential TPE‐IVIG‐cyclosporine A represents a feasible pragmatic option in selected cases when standard regimens are inaccessible, though causality cannot be established from a single case with multiple concurrent interventions.
5. In adult HLH, malignancy‐associated aetiologies—particularly SPTCL, IVLBCL, and AITL—must be systematically considered, as management approaches differ fundamentally from autoimmune‐associated HLH.
6. Panniculitis plaques and ulcers in this context represent a spectrum of the same pathological process at different stages of disease evolution, not distinct entities.

## 8 Patient Perspective

The long fevers, repeated admissions and painful leg ulcers were frightening and exhausting. I was worried about losing my ability to walk or being left with permanent damage. Once the diagnosis was explained in simple words and I began to see the ulcers healing and my strength returning, my anxiety decreased. Regular blood tests and cyclosporine monitoring now feel like a small price to pay for stability and a more normal life. I agreed to share my story so that other patients with rare inflammatory diseases might benefit.

## Author Contributions

**Hatem Mousa Taha:** conceptualization, data curation, formal analysis, investigation, methodology, supervision, writing – original draft, writing – review and editing. **Omar Marouf:** data curation, resources, writing – review and editing. **Khaled Hatem Taha:** formal analysis, investigation, writing – review and editing.

## Funding

The authors have nothing to report.

## Disclosure

CARE compliance: This case is reported according to CARE guidelines [8].

## Ethics Statement

According to institutional policies, formal research ethics committee approval is not required for anonymized single‐patient case reports.

## Consent

Written informed consent was obtained from the patient for publication of her clinical details and associated images.

## Conflicts of Interest

The authors declare no conflicts of interest.

## Supporting information


**Table S1:** Structured differential diagnosis considered in this case.

## Data Availability

De‐identified data underlying this report are available from the authors upon reasonable request.

## References

[1] L. Fardet , L. Galicier , O. Lambotte , et al., “Development and Validation of the HScore, a Score for the Diagnosis of Reactive Hemophagocytic Syndrome,” Arthritis & Rhematology 66, no. 9 (2014): 2613–2620. 10.1002/art.38690 24782338

[2] P. La Rosée , A. Horne , M. Hines , et al., “Recommendations for the Management of Hemophagocytic Lymphohistiocytosis in Adults,” Blood 133, no. 23 (2019): 2465–2477. 30992265 10.1182/blood.2018894618

[3] M. Ramos‐Casals , P. Brito‐Zerón , A. López‐Guillermo , M. A. Khamashta , and X. Bosch , “Adult Haemophagocytic Syndrome,” Lancet 383, no. 9927 (2014): 1503–1516. 24290661 10.1016/S0140-6736(13)61048-X

[4] G. S. Schulert and A. A. Grom , “Macrophage Activation Syndrome and Cytokine‐Directed Therapies,” Best Practice & Research. Clinical Rheumatology 36, no. 3 (2022): 101773. 24974063 10.1016/j.berh.2014.03.002 PMC4074772

[5] D. T. Lounder , Q. Bin , C. de Min , and M. B. Jordan , “Treatment of Refractory Hemophagocytic Lymphohistiocytosis With Emapalumab Despite Severe Concurrent Infections,” Blood Advances 3, no. 1 (2019): 47–50. 30617216 10.1182/bloodadvances.2018025858 PMC6325304

[6] A. Ahmed , S. A. Merrill , F. Alsawah , et al., “Ruxolitinib in Adult Patients With Secondary Haemophagocytic Lymphohistiocytosis: An Open‐Label, Single‐Centre, Pilot Trial,” Lancet Haematology 6, no. 12 (2019): e630–e637. 31537486 10.1016/S2352-3026(19)30156-5 PMC8054981

[7] A. M. Schram and N. Berliner , “How I Treat Hemophagocytic Lymphohistiocytosis in the Adult Patient,” Blood 125, no. 19 (2015): 2908–2914. 25758828 10.1182/blood-2015-01-551622

[8] J. J. Gagnier , G. Kienle , D. G. Altman , et al., “The CARE Guidelines: Consensus‐Based Clinical Case Reporting Guideline Development,” Global Advances in Health and Medicine 2, no. 5 (2013): 38–43. 10.7453/gahmj.2013.008 PMC3833570 24416692

[9] E. Karakike , E. J. Giamarellos‐Bourboulis , C. Mikacenic , et al., “Respiratory Failure and the Hyperferritinemia Syndrome,” Critical Care 25 (2021): 341.34535154

[10] A. Gupta , P. Tyrrell , R. Valani , S. Benseler , S. Weitzman , and M. Abdelhaleem , “The Role of the Initial Bone Marrow Aspirate in the Diagnosis of Hemophagocytic Lymphohistiocytosis,” Pediatric Blood & Cancer 51, no. 3 (2008): 402–404. 18523990 10.1002/pbc.21564

[11] J. I. Henter , A. Horne , M. Aricò , et al., “HLH‐2004: Diagnostic and Therapeutic Guidelines for Hemophagocytic Lymphohistiocytosis,” Pediatric Blood & Cancer 48, no. 2 (2007): 124–131. 16937360 10.1002/pbc.21039

[12] T. Gayden , F. E. Sepulveda , D. A. Khuong‐Quang , et al., “Germline HAVCR2 Mutations Altering TIM‐3 Characterize Subcutaneous Panniculitis‐Like T‐Cell Lymphoma With Hemophagocytic Lymphohistiocytosis,” Nature Genetics 50, no. 12 (2018): 1650–1657. 30374066 10.1038/s41588-018-0251-4

[13] C. Polprasert , Y. Takeuchi , N. Kakiuchi , et al., “Frequent Germline Mutations of HAVCR2 in Subcutaneous Panniculitis‐Like T‐Cell Lymphoma,” Blood Advances 3, no. 4 (2019): 588–595. 30792187 10.1182/bloodadvances.2018028340 PMC6391671

[14] Q. Zhang , Y. He , N. Luo , et al., “Ruxolitinib for Subcutaneous Panniculitis‐Like T‐Cell Lymphoma With HAVCR2 Mutation: Targeting Immune Checkpoint–Driven Hyperinflammation,” Journal of Clinical Investigation 131, no. 21 (2021): e149136.

[15] D. Demirkol , D. Yildizdas , B. Bayrakci , et al., “Therapeutic Plasma Exchange in the Treatment of Secondary Hemophagocytic Lymphohistiocytosis: A Multicentre Study,” Pediatric Critical Care Medicine 23, no. 3 (2022): e147–e154.

[16] M. C. Bunte and M. M. Patnaik , “Use of Plasma Exchange in Hemophagocytic Syndromes,” Transfusion and Apheresis Science 52, no. 2 (2015): 139–146.

[17] K. Azushima , M. Ohsawa , S. Watanabe , et al., “Effectiveness of Double Filtration Plasmapheresis in Multidrug‐Resistant Adult Hemophagocytic Syndrome,” Therapeutic Apheresis and Dialysis 23, no. 3 (2019): 249–255.

[18] R. Reshef , S. M. Luger , and E. O. Hexner , “Therapeutic Plasma Exchange and Intravenous Immunoglobulin for the Treatment of Hemophagocytic Lymphohistiocytosis,” Blood Reviews 28, no. 5 (2014): 201–211.

[19] M. Gerfaud‐Valentin , Y. Jamilloux , J. Iwaz , and P. Sève , “Adult‐Onset Still's Disease,” Autoimmunity Reviews 13, no. 7 (2014): 708–722. 24657513 10.1016/j.autrev.2014.01.058

[20] A. Ravelli , A. A. Grom , E. M. Behrens , and R. Q. Cron , “Macrophage Activation Syndrome as Part of Systemic Juvenile Idiopathic Arthritis: Diagnosis, Genetics, Pathophysiology and Treatment,” Genes and Immunity 13, no. 4 (2012): 289–298. 22418018 10.1038/gene.2012.3

[21] C. E. Allen , X. Yu , C. A. Kozinetz , and K. L. McClain , “Highly Elevated Ferritin Levels and the Diagnosis of Hemophagocytic Lymphohistiocytosis,” Pediatric Blood & Cancer 50, no. 6 (2008): 1227–1235. 18085676 10.1002/pbc.21423

