# Supplementary material for: Severe Adult HLH/MAS With SPTCL‐Like Panniculitis: A Phenotype‐Guided, Resource‐Adapted Therapeutic Strategy Without Cytotoxic Therapy
Source: Clin Case Rep. 2026 Apr 29;14(5):e72638. doi: 10.1002/ccr3.72638 (PMC13128521; doi:10.1002/ccr3.72638)
Supplement: Supplementary file 1 — Table S1: Structured differential diagnosis considered in this case. [file CCR3-14-e72638-s001.docx]

**Supplementary Table S1. Structured differential diagnosis considered in this case**

**Legend:**
Differential diagnostic considerations evaluated during assessment of this patient with severe hyperferritinaemic inflammation, including clinical features supporting or arguing against each diagnosis and key investigations used to refine diagnostic probability. The table highlights the rationale for favouring secondary autoimmune-associated HLH/MAS in this case.

| Condition | Supporting features | Features against / less supportive | Key tests / comments |
| --- | --- | --- | --- |
| Adult-onset Still's disease with MAS | Hyperferritinaemia; fever; hepatosplenomegaly; cytopenias | No evanescent rash, inflammatory arthritis, or pharyngitis; chronic neutrophilic panniculitis predating flare; autoimmune serologies negative | AOSD remains a clinical diagnosis; absence of typical triad reduces likelihood but does not exclude. Consider serial clinical reassessment if relapse. |
| Infection-driven cytokine storm / severe sepsis | Necrotic ulcers with Pseudomonas; fever; inflammatory response | Blood cultures repeatedly sterile; documented microbiological clearance with persistent hyperferritinaemia and hypofibrinogenaemia at peak activity | Continue to prioritise source control and targeted antimicrobials; interpret kinetics in parallel with infection markers and cultures. |
| Malignancy-associated HLH | HLH phenotype possible in adults | CT chest/abdomen/pelvis showed hepatosplenomegaly without lymphadenopathy or masses; bone marrow without malignancy | Repeat evaluation if relapse or new red flags; consider PET-CT if available. |
| Primary (genetic) HLH / monogenic autoinflammation | Recurrent inflammatory skin disease could indicate predisposition | Adult onset; no family history; durable remission without etoposide | Genetic testing not available; acknowledge as limitation; reconsider if recurrent episodes or atypical course. |
| VEXAS syndrome | Adult hyperinflammation can mimic HLH/MAS | Predominantly older men; typical macrocytic anaemia, chondritis, and marrow vacuoles not observed; young female patient | Definitive exclusion requires UBA1 testing (not available). Female cases are rare; consider if macrocytosis/chondritis develop. |
